# Mandevillian intelligence

**DOI:** 10.1007/s11229-017-1414-z

**Published:** 2017-05-17

**Authors:** Paul R. Smart

**Affiliations:** Electronics and Computer Science, University of Southampton, Highfield, Southampton, SO17 1BJ UK

**Keywords:** Collective cognition, Group cognition, Collective intelligence, Social simulation, Social epistemology, Cognitive technology

## Abstract

Mandevillian intelligence is a specific form of collective intelligence in which individual cognitive vices (i.e., shortcomings, limitations, constraints and biases) are seen to play a positive functional role in yielding collective forms of cognitive success. The present paper introduces the concept of mandevillian intelligence and reviews a number of strands of empirical research that help to shed light on the phenomenon. The paper also attempts to highlight the value of the concept of mandevillian intelligence from a philosophical, scientific and engineering perspective. Inasmuch as we accept the notion of mandevillian intelligence, then it seems that the cognitive and epistemic value of a specific social or technological intervention will vary according to whether our attention is focused at the individual or collective level of analysis. This has a number of important implications for how we think about the design and evaluation of collective cognitive systems. For example, the notion of mandevillian intelligence forces us to take seriously the idea that the exploitation (or even the accentuation) of individual cognitive shortcomings could, in some situations, provide a productive route to collective forms of cognitive and epistemic success.

## Introduction

Issues of collective cognition^1^ and collective intelligence have recently emerged as a common focus of interest across a broad array of disciplines. The cognitive properties of social groups and socio-technical systems have, of course, been a focus of long-standing interest for disciplines such as cognitive science (Hutchins 1995), social psychology (Hinsz et al. 1997; Kerr and Tindale 2004), human factors (Cooke et al. 2007), the philosophy of mind (Theiner 2014; Theiner et al. 2010) and social/collective epistemology (Goldman and Whitcomb 2011; Lackey 2014; Brady and Fricker 2016). Recently, however, there has been a burgeoning of research interest into issues of collective cognition within the computational, information and network sciences. Within computer science, for example, we have seen the emergence of research into issues of social computation (Kearns 2012), collective intelligence (Malone and Bernstein 2015), augmented social cognition (Chi et al. 2008), socio-computational systems (Mason 2013; Michelucci 2013), crowdsourcing (Michelucci and Dickinson 2016; Bigham et al. 2015), social machines (Hendler and Berners-Lee 2010; Smart and Shadbolt 2014) and the global brain (Heylighen 2013). In general, what is common to all these areas of research is an interest in the factors that enable groups of individuals to coordinate their collective efforts in support of specific cognitive or epistemic objectives. There is, in other words, a common interest in the factors that support the emergence of intelligent behavior at the collective or social level of analysis.

Relative to the goal of supporting collective cognitive success, the properties of individual cognitive agents are often seen as somewhat problematic. There is thus a substantial literature that highlights the various things that can go awry with collective cognizing. Sunstein (2011) provides a useful summary of some of the more common worries relating to groupthink (Janis 1982), group polarization (Myers and Lamm 1976), hidden profiles (Stasser and Titus 2003), the common knowledge effect (Gigone and Hastie 1993) and information cascades (Sunstein 2011). All these phenomena highlight the problematic status of groups as the effective processors of information (see Hinsz et al. 1997), and they reveal a concern about the relationship between individual, agent-level cognitive properties and their affect on collective cognitive outcomes. One expression of this concern is that the cognitive properties of individual agents often work against the interests of the collective, vis-à-vis the realization of intelligent outcomes. It is well-established, for example, that at least some forms of individual cognitive bias can be accentuated at the group or collective level (Hinsz et al. 2008; Hutchins 1991).

The present paper attempts to provide a contrasting vision of the relationship between individual, agent-level properties and the emergence of collective intelligence. In particular, the central claim of this paper is that individual cognitive shortcomings can, on occasion, play a productive role in supporting collective forms of cognitive success. This idea is captured by the notion of mandevillian intelligence:

**Mandevillian Intelligence**

Cognitive and epistemic properties that are typically seen as shortcomings, limitations or biases at the individual level can, on occasion, play a positive functional role in supporting the emergence of intelligent behavior at the collective level.

Thus conceived, mandevillian intelligence is identified as a specific form of collective intelligence. It is, however, a form of collective intelligence that is predicated on a particular category of individual-level properties, *viz.*, those properties of individual agents that are typically denigrated as cognitive shortcomings or ‘cognitive vices’. Such vices include a range of cognitive constraints, limitations and biases, as well as an assortment of intellectual character traits, such as those discussed by proponents of character-based virtue epistemology (see Zagzebski 1996; Baehr 2008, 2012).^2^ All these cognitive vices can, I suggest, contribute to the emergence (realization?) of properties that are often construed as ‘virtues’ at the collective level. By this I mean that individual-level cognitive shortcomings, limitations or biases can play a productive role in bringing about a set of desirable cognitive properties at the group, social or collective level. This includes, most notably, the cognitive and epistemic properties that we typically associate with good performance in collective cognitive processing contexts, e.g., the ability to make good decisions, the ability to form correct interpretations, and the ability to engage in truth-conducive forms of reasoning. It also includes, although I will not discuss this in the present paper, the properties that enable groups to form and function in the first place. Here, there has been some intriguing work regarding the role of individual mnemonic shortcomings, such as forgetting and distortion, in enhancing sociality (Brown et al. 2012; Fagin et al. 2013) and supporting the emergence of cooperative behaviors (Horváth et al. 2012).

The concept of mandevillian intelligence may be seen to be in some tension with much of the existing work in collective cognition and collective intelligence. This is because individual cognitive shortcomings are, more often than not, seen to pose a threat to collective forms of cognitive success. The concept of mandevillian intelligence also promises (or perhaps threatens) to challenge our views regarding the cognitive or epistemic value of interventions that seek to enhance (and sometimes undermine^3^) various aspects of individual-level cognitive functioning. It is for these reasons, I suggest, that the notion of mandevillian intelligence marks out an important area of theoretical and empirical interest for a broad array of academic disciplines.

The aim of the present paper is to review a number of strands of cognitive scientific research that provide a degree of evidential support for the notion of mandevillian intelligence. The paper also seeks to highlight, at least in summary form, the value of the concept of mandevillian intelligence. The status of mandevillian intelligence as a specific form of collective intelligence is discussed in Sect. 2. This section also clarifies the meaning of the terms “vice” and “virtue,” at least as they are used in the remainder of the paper. Sections 3 and 4 are intended to highlight the basic plausibility of mandevillian intelligence with respect to the mechanisms that underpin the cognitive performance of groups, teams and other multi-agent ensembles. In particular, Sect. 3 discusses the role of individual, agent-level cognitive properties in configuring the performance-relevant structural profile of a communication network. Such configurational changes, it is suggested, provide insight in the nature of the causal mechanisms that could underlie mandevillian intelligence. With an understanding of such causal mechanisms to hand, it should be clear that there is nothing particularly ‘mysterious’ about mandevillian intelligence: if we accept that individual-level cognitive shortcomings can alter the dynamics of information processing within a collective, multi-agent system, then it is perfectly possible (at least in principle) that some of those shortcomings will alter information processing in the sort of way that is required for collective forms of cognitive success.

Subsequent sections of the paper are intended to convince the reader that we should take the notion of mandevillian intelligence seriously. The role of individual cognitive vices in yielding collective forms of cognitive success is thus discussed in relation to dogmatism (Sect. 4), limited working memory (Sect. 5), forgetting (Sect. 6) and confirmation bias (Sect. 7). Finally, Sect. 8 highlights the value of the concept of mandevillian intelligence with respect to a range of issues that lie at the heart of contemporary debates in computer science, cognitive science, epistemology and the philosophy of mind.

## Vice, virtue and mandevillian intelligence

The concept of mandevillian intelligence is named after the Anglo-Dutch philosopher, political economist and satirist, Bernard Mandeville. As should be clear from the quote at the beginning of the present paper, Bernard Mandeville was concerned with the relationship between private (individual) vice and public (collective) benefit. In particular, Mandeville suggested that individual vice could, under the right conditions, lead to economic prosperity. The concept of mandevillian intelligence reflects an attempt to transpose this idea to the cognitive domain. In particular, mandevillian intelligence is cast as a particular form of collective intelligence in which individual, agent-level cognitive shortcomings, limitations or biases (vices) are seen to play a productive role in enhancing the cognitive performance of a team, group or some other collective. Mandevillian intelligence is, in essence, the idea that the cognitive vices of the one (the individual) are causally linked to the cognitive virtues of the many (the collective).

It is important to be clear what is (and what is not) meant by the term “mandevillian intelligence.” Firstly, it is important to emphasize that mandevillian intelligence is, for the purposes of the present paper, cast as a specific form of *collective intelligence*. For this reason, the concept of mandevillian intelligence is not applicable to situations in which individual cognitive shortcomings, limitations or biases lead to negative (non-intelligent) outcomes at the collective level. This is because such situations do not count as *bona fide* cases of collective intelligence—they are perhaps best seen as instances of ‘collective stupidity’ or as failures of collective intelligence. The notion of mandevillian intelligence is thus not impugned by evidence regarding the negative effect of vice-like cognitive properties (e.g., individual forms of cognitive bias) on collective performance (see Hinsz et al. 2008).

Secondly, it is important to note that the concept of mandevillian intelligence does not see individual cognitive vice as necessary for *all* forms of collective intelligence. It is thus perfectly possible that collective intelligence could be exhibited in the absence of individual vice. As a result, mandevillian intelligence should not be mistaken as the view that all forms of individual vice are beneficial for collective performance, or that all cases of collective intelligence are grounded in the presence of individual cognitive vice.

It is also important to be clear about the meaning of terms such as “vice” and “virtue,” again as they are used in the present paper. In a general sense, the term “cognitive vice” is used to refer to a cognitive property that is typically denigrated as a shortcoming, limitation or bias. A poor memory (e.g., failure to recall), for example, might be regarded as a vice, while a perfect memory (e.g., perfect recall) might be regarded as a virtue. Vices and virtues, in this sense, are evaluated with respect to performance-related criteria, with higher levels of performance judged as ‘good’ or desirable.^4^

The point of mandevillian intelligence is to encourage a degree of scepticism with respect to the perceived value of interventions that aim to ‘enhance’ individual levels of cognitive performance (e.g., interventions that improve attentional capacity or mnemonic recall). In particular, mandevillian intelligence encourages us to consider the possibility that a seemingly sub-optimal aspect of individual cognitive functioning could play a role in yielding collective forms of cognitive success. Mandevillian intelligence thus extends the familiar idea that individual cognitive limits may have adaptive value for the individual (Bjorklund 1997; Hertwig et al. 2003; Kareev 2012; Lungarella and Berthouze 2002) to the realm of collective cognizing. It is, in essence, the idea that enhanced levels of cognitive performance at the collective level can stem from seemingly sub-optimal levels of cognitive performance at the individual level.

As a means of helping us get a better understanding of mandevillian intelligence, consider the results of recent work relating to the so-called “Wisdom of the Inner Crowd” (Rauhut and Lorenz 2011). The Wisdom of the Inner Crowd phenomenon is the individual counterpart to the conventional “Wisdom of the Crowds” phenomenon (Surowiecki 2005), wherein independent judgements from multiple individuals are combined to yield an aggregate response. In the case of the Wisdom of the Inner Crowd, a single individual is asked to make a series of judgements, and these judgements are then combined to form a ‘collective’ output. Interestingly, Hourihan and Benjamin (2010) report that the Wisdom of the Inner Crowd is enhanced in subjects with shorter memory spans. In other words, the results of Hourihan and Benjamin (2010) suggest that a specific cognitive shortcoming (i.e., limited memory span) enhances individual performance on a specific cognitive task. All that the concept of mandevillian intelligence seeks to do is to extend this sort of idea to the realms of collective (as opposed to individual) performance.

Empirical support for the notion of mandevillian intelligence comes from a variety of quarters. As will be evident from the studies reviewed in subsequent sections, much of the currently available research relies on the use of computer simulation techniques. There is, however, evidence to suggest that mandevillian intelligence is a phenomenon that applies to real-world collective organizations, such as human groups, teams and communities. Solomon (1992), for example, appeals to a form of mandevillian intelligence when he suggests that “cognitive bias and belief perseverance on the part of geologists during the geological revolution was, contrary to what might be expected, in fact conducive to scientific success” (p. 443). There is even evidence to suggest that mandevillian-like mechanisms may underlie the forms of collective intelligence exhibited by eusocial insects (Weidenmüller and Seeley 1999; Dussutour et al. 2009). There is, as such, every reason to think that the concept of mandevillian intelligence helps to identify a phenomenon that is shared by a rich array of materially-disparate collective organizations, ranging from collections of purely synthetic cognitive agents, through to human teams and animal communities.

## Collective search

At first sight, it might be thought that claims about mandevillian intelligence are highly implausible. How could a seemingly defective or sub-optimal form of cognitive functioning at the individual level contribute to the collective intelligence of a community? As a means of addressing this ‘plausibility issue’, it will help to have a better understanding of the mechanisms that contribute to the performance of multi-agent collectives. The present section thus seeks to highlight the basic plausibility of mandevillian intelligence with respect to the way in which individual, agent-level cognitive properties can dynamically influence the flow of information and influence with a collective, multi-agent ensemble.

Many real-world problems require the cooperative effort of multiple individuals, and the nature of the communication that takes place between such individuals is often key to understanding their collective successes (and failures). But how should the efforts of a group be organized so as to optimize their chances of collective success? How, in particular, should the flow of information and influence within a group be structured (in both space and time) so as to ensure the group exhibits collective intelligence?

One way of answering this question is to compare the performance of groups (or, more generally, agent collectives) in situations where the structure of the agent communication network is systematically varied. A number of such studies have been undertaken with both human subjects (Judd et al. 2010; Kearns 2012; Kearns et al. 2006; Mason 2013; Mason and Watts 2012; Mason et al. 2008) and synthetic agents (Lazer and Friedman 2007; Reitter and Lebiere 2012; Smart et al. 2010; Xu et al. 2016). As is noted by Mason (2013), many of the tasks used in these studies can be usefully viewed as a form of collective search task. In other words, the nature of the task being performed by the collective ensemble can be understood as an attempt to explore a space of possible solutions, where the value of specific solutions is indicated by some form of objective function.^5^ This, I suggest, is indeed a useful way of thinking about research into collective cognition. One benefit of such a view is that it helps us to interpret the effects of a variety of experimental manipulations in terms of what has come to be known as the trade-off between exploration and exploitation (March 1991).^6^ Achieving an effective balance between exploration and exploitation is important to many forms of collective endeavor, and it lies at the heart of recent attempts to deliberately structure the dynamics of information flow in a way that helps to improve collective outcomes (Pentland 2013, 2014). A second benefit of conceptualizing collective cognition as a form of collective search is that we are able to identify a potentially productive linkage with research into search optimization (Burke and Kendall 2010). This turns out to be particularly important in the case of what is called particle swarm optimization (Poli et al. 2007). In fact, many of the simulation experiments in particle swarm optimization are similar to those encountered in the case of computational studies of collective cognition.

One study that has sought to examine collective cognition as a form of collective search is a study by Mason et al. (2008). Mason et al. were interested in the ability of groups of networked human subjects to collectively explore a problem space and find optimal solutions within that space. In particular, the subjects in Mason et al.’s study had to choose (or guess) a number between 0 and 100, and they were awarded points based on a score associated with the selected number. A continuous fitness function (a form of objective function) was used to determine the score associated with each number, but this function was not made available to the subjects performing the task. Instead, on any given round of the experiment, the subjects had to choose a number based on the feedback they received from both their own guesses and the guesses of their immediate network neighbors. Collective problem-solving performance was then assessed by calculating the average score of all the subjects on each round of the experiment. In order to achieve success on this task, subjects therefore had to explore the structure of the fitness landscape, as determined by the fitness function, and then converge on the most optimal solution (i.e., number) available. The cognitive process in question is thus a form of collective problem-solving in which the search efforts of multiple individuals are pooled to create a measure of collective cognitive success (i.e., the ability of the group to find an optimal solution).

**Fig. 1:**
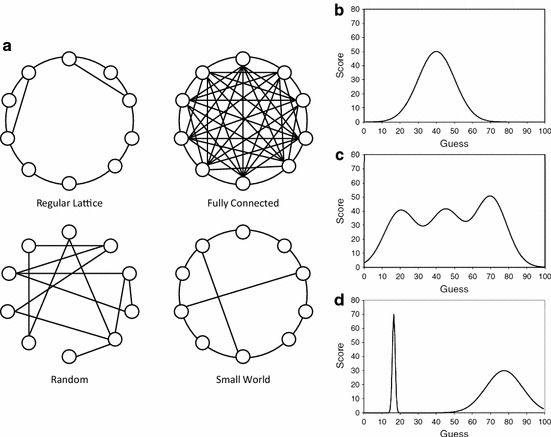
Example network structures and problem spaces studied by Mason et al. (2008). **a** Examples of the kinds of communication network structures used by Mason et al. (2008). **b** Unimodal problem space. **c** Multi-modal problem space. **d** Needle problem space

In order to assess the effect of network structure on problem-solving performance, Mason et al. organized the human subjects into communication networks with different structural topologies (see Fig. 1a). In the fully connected network, each participant could see the solutions (and associated fitness scores) of all the other participants. In the case of other networks, however, each participant could only see the solutions and scores of a more limited number of individuals, namely their immediate network neighbors. The question that can now be asked is: How will the structure of the communication network influence the collective problem-solving behavior (and performance) of the participants? How, in other words, will network structure affect the problem-solving performance of the human groups?

What Mason et al. found was that in conditions in which there was a single optimum (i.e., a fitness landscape with a single peak) (see Fig. 1b), the network structures that supported the most rapid dissemination of information were the most successful. Thus, when the fitness landscape had a single peak, subjects tended to converge more quickly on the global maximum in the fully connected, small world and random network topology conditions. This result can be understood in terms of the speed at which information propagates through these networks. Thus, with a single peak, each solution with a higher score provides information about the best direction for future search efforts (all routes uphill lead to the same optimal solution). As a result, it is helpful to have a situation whereby information about the results of collective search efforts are distributed as widely as possible throughout the problem-solving community—this produces the greatest efficiency in finding the globally optimal solution.

Things are very different, however, when the fitness landscape has a more rugged, multi-modal structure, i.e., when there are multiple local optima (and one global optimum) (see Fig. 1c). In this case, we might expect rapid information dissemination to once again result in rapid convergence on a particular solution; however, whether that solution is the best one available (i.e., whether it is the globally-optimal solution) will depend on how lucky the participants are with respect to their initial guesses. If the participants refrain from converging too quickly on a particular solution, they might discover a more globally-optimal solution, and it is for this reason that we might expect network structures that limit or retard the rate of information dissemination to benefit collective search efforts.

This is pretty much what Mason et al. (2008) discovered. They found that when a multi-peaked solution landscape was used (see Fig. 1c), participants found the global solution fastest in the small-world network condition. The topology of the small-world network, in this case, seems to provide just the right amount of social influence for optimal performance: it supports a certain amount of independent exploration, but it does not rule out the possibility of rapid convergence on high-scoring solutions.

In another multi-peaked landscape, where the globally-optimal solution was to found at the apex of a sharply-defined elevation in the landscape (a ‘needle’ problem space) (see Fig. 1d), another type of network—a lattice network—tended to yield the best overall performance. The explanation for this result relates to the fact that the lattice network has the longest path length and thus retards the rate of information propagation. This prevents individuals from being drawn towards an attractive, albeit sub-optimal, solution before the path to the globally-optimal solution has been discovered.

Results similar to Mason et al. (2008) have been reported by Lazer and Friedman (2007), this time using computer simulation techniques. As with Mason et al., Lazer and Friedman examined the effect of network structure on collective problem-solving performance using a search task that featured fitness landscapes of varying complexity. At the beginning of each simulation, agents were randomly assigned to a location on the fitness landscape (where each location corresponded to a candidate solution). Then, on each round of the simulation, the agents were able to explore the immediate vicinity of the solution landscape and move to better locations (i.e., adopt a fitter candidate solution). In addition to receiving information about the fitness of their own solutions, agents also received information about the fitness of solutions as discovered by their network neighbors. If a neighboring agent proposed a solution that was fitter than the agent’s own solution, then they adopted the solution of the neighboring agent.

What Lazer and Friedman discovered was a trade-off in the way in which different network structures established a productive balance between exploration and exploitation. Exploration, in this case, is the tendency of agents to explore the solution space independently of other agents; exploitation, in contrast, is the tendency of agents to adopt the solutions proposed by other agents. The need to balance these tendencies resulted in a profile of topology-dependent performance in which the networks with low average path lengths (e.g., fully connected networks) yielded better performance in the short-term—relative to networks with higher average path lengths (e.g., linear networks)—but worse performance in the longer term—again relative to networks with higher average path lengths.

The results of studies exploring the effect of network structure on collective search thus point towards a common conclusion: different types of communication network topology can affect the rate at which information propagates within a problem-solving community and this influences the group’s ability to discover globally-optimal solutions. This seems to be especially so in situations where early successes are of little value in terms of illuminating the path to the best outcome. In particular, as the structure of the solution landscape becomes progressively more rugged, and the complexity of the target problem becomes correlatively more complex, the rate of information dissemination seems to become increasingly important in determining the success of collective search efforts. Networks that facilitate the rapid transfer of information between the members of a problem-solving community can, it seems, result in precipitant forms of information sharing, especially on more complex problem types (as represented by more rugged solution landscapes). In the worst case, this can promote premature convergence on sub-optimal solutions and thus derail the efforts of the community to investigate alternative (and more profitable) parts of the solution space.

Such insights are useful in helping us see how mandevillian intelligence might be realized. For what seems to be crucial in the case of collective search is that there is some degree of adaptive alignment between the complexity of the search problem and the way in which agents are influenced by the flow of information within a communication network. The studies by Lazer and Friedman and (to a lesser extent) Mason et al. assume that individual agents will always adopt the superior solutions of their network neighbors, and thus the structure of the communication network emerges as the primary factor that shapes collective performance. In real-world problem-solving situations, however, a variety of forces and factors may conspire to influence the rate at which information flows through a communication network, as well as the extent to which agents are prepared to adopt or embrace the solutions of their network neighbors. The properties of individual agents can thus, at least to some extent, regulate what is sometimes called the *effective connectivity* of a communication network (see Sect. 4). An individual agent may, for example, refuse to heed the solutions proposed by other agents, or they may refuse to communicate the results of their own search efforts on account of their desire to be the one who discovers the ultimate solution to some problem. Here, then, we can begin to see how private ‘vices’ can sometimes contribute to the public good: by limiting the flow of information through a community, agents can sometimes change the effective structure of a communication network in ways that help to preserve diversity and prevent (premature) convergence on sub-optimal solutions.

In human social networks, there are clearly a variety of cognitive factors that might play this sort of functional role.^7^ They include the tendency to hoard information, a lack of willingness to cooperate with others, a vulnerability to copying/transmission errors,^8^ and a steadfast (and seemingly irrational) commitment to revise one’s own ideas in the light of seemingly superior alternatives. Importantly, for present purposes, these factors are often cast in a somewhat negative light: they are often seen as agential properties that need to be ameliorated by some form of social or technological intervention. The notion of mandevillian intelligence, however, gives us another way of looking at these individual ‘vices’. In particular, it enables us to see how individual cognitive vices might work to control the dynamics of information flow and influence within a community, enabling the community to deal effectively with the challenges posed by certain kinds of problem.

## Distrust, dogmatism and dynamic networks

On the basis of the results reported by Mason et al. (2008) and Lazer and Friedman (2007), we might be drawn to the conclusion that different types of network structure are differentially suited to different types of search problem. In situations where the solution landscape is highly rugged (e.g., a mountainous terrain with soaring cliffs and sheer drops) it thus seems that networks with the longest path lengths are likely to yield the best overall performance. However, in situations where the solution landscape is somewhat simpler (e.g., a single hillock surrounded by a flat plain), networks with the shortest path lengths (i.e., fully connected networks) seem to fit the bill. This conclusion, however, overlooks the possibility that different types of network structure may be differentially effective at different *stages* of a collective problem-solving process. Thus, even when we are confronted with a highly rugged solution landscape, this does not mean that we should always countenance slow rates of information transmission for the entire duration of the task. A better way to organize collective search efforts may be to limit inter-agent communication at the outset of the search process and then encourage greater levels of communication (and thus influence) as the search progresses. As noted by Zollman (2010), even in cases where some degree of epistemic or cognitive diversity is deemed to be a virtue, we seldom want to preserve diversity throughout the entire duration of the collective search process. At some point a consensus should be reached, or the community should, at least, focus its efforts on a more restricted part of the search space, namely, that region where the globally-optimal solution is to be found. What we have, then, is the idea of a kind of stage-dependent search effort, one in which initial diversity and independent exploration are substituted (at later stages) for convergence and mutual exploitation.

One way of applying this idea to the study of collective cognitive systems is to think about dynamic changes in the structure of the communication network that is used to support the exchange of information. In other words, a key parameter of interest when it comes to the study of collective cognition is the structural organization of a network’s topology *across time*. In essence, we are interested in how the pattern of linkages between nodes in some form of networked ensemble (e.g., a social or neural network) emerge (and fluctuate) across the course of some cognitive task. There are a variety of reasons to think that such time-variant changes in the structural organization of a networked system might yield performance benefits. In the case of machine learning, for example, it has been suggested that learning outcomes can be improved by enabling neural networks to dynamically alter their organization by, for example, acquiring additional nodes (see Ash 1989). In addition, techniques that reduce network size have been shown to have a positive impact on network performance, at least in some training contexts. The progressive elimination of nodes from the hidden unit layer of neural networks thus forces a neural network to learn a more concise characterization of the regularities underlying successful performance in a given problem domain (Mozer and Smolensky 1989a, b). Dynamic changes in network structure are also relevant to neuroscientific accounts of cognition. The neural selectionist account of Edelman (1987), for instance, maintains that cognitive development is informed by an initial activity-independent overproduction of synaptic resources followed by a period of activity-dependent synaptic selection and pruning.

In spite of all this, there is relatively little research that seeks to investigate the role of dynamic networks in shaping the performance profile of collective (i.e., multi-agent) cognitive systems. This is unfortunate, since dynamic networks are clearly the norm when it comes to many real-world cases of collective cognition (see Mason et al. 2007).

One study that has sought to examine the impact of dynamic networks on collective performance is a study by Smart et al. (2010). In this case, the performance profiles of static and dynamic communication networks were compared in a collective search task identical to that used by Lazer and Friedman (2007). The dynamic networks used in the study were a form of incrementally constructive network, in which inter-agent communication links were progressively added (at random) to the agent community until all the constituent agents were connected into a single network component. Using this approach, Smart et al. (2010) showed that dynamic networks yielded better levels of collective search performance as compared to their more statically-configured counterparts. It thus appears that dynamic networks may help to produce an effective balance between exploration and exploitation—a balance that (in this case) depends on the time-variant structural dynamics of the networks used to support the socially-mediated exchange of task-relevant information.

In some ways, this conclusion should not come as a surprise, especially to those who work in the areas of swarm intelligence and search optimization. A common element of many search-oriented multi-agent systems is thus the use of a dynamically varying parameter that serves to guide the larger systemic organization towards some optimal outcome. In the case of swarm intelligence, for example, it has long been known that foraging ants rely on pheromone trails as a means of guiding their collective responses to available food sources (Garnier et al. 2007). An important characteristic of such trails is that they are subject to use-dependent forms of signal decay and amplification. As some trails are progressively amplified as a result of scent deposition, other trails invariably weaken as a result of scent dissipation. The result is an adaptive alignment of food gathering assets (i.e., individual ants) with respect to the relative profitability of different food sources within the local environment.^9^

Time-dependent changes in system control parameters are also encountered in the context of research into search optimization. Consider, for example, the case of simulated annealing (Kirkpatrick et al. 1983). Simulated annealing is a search optimization technique that relies on a gradual decrease in ‘thermal’ energy as a means of striking a productive balance between initial exploration and subsequent exploitation. Similar time-dependent trade-offs in exploration and exploitation can be found in the case of particle swarm optimization (Poli et al. 2007). The original particle swarm algorithm, as proposed by Kennedy and Eberhart (1995), for instance, relies on the use of individual agents (particles) to explore a solution space and converge on a global optimum. A key feature of the algorithm is that the agents are drawn to the best location they have found as well as the best location found by other members of the collective. As noted by Mason (2013), the challenge confronting this algorithm is to ensure an effective trade-off between exploration and exploitation. This, he suggests, can be controlled by the incremental adjustment of parameters that progressively increase the attractiveness of particular parts of the solution landscape across the duration of the search task.

There is thus a common theme that permeates the literature on collective search. This is the idea that one or more *dynamic* system parameters can be used to good effect in terms of regulating the balance between exploration and exploitation. Structural changes in network topology are obviously one way of achieving this regulation. However, they are by no means the only way. Crucially, we should distinguish between a number of different forms of connectivity when it comes to the analysis of network systems (see Friston 2011). One of these forms of connectivity is the structural connectivity of the network—the physical linkages that enable nodal elements to communicate with one another. Another is the effective connectivity of the network—the linkages that are actively involved in mediating the flow of information and influence across the networked organization at a particular point in time. These two kinds of connectivity are not the same (although they are easily confused), and they often lead to very different topological characterizations of what is, in effect, the same network system. Crucially, when it comes to task performance, it is often effective connectivity, rather than structural connectivity, that counts. Consider, for example, the human brain. The human brain can clearly be characterized in terms of its hodological structure—the pattern of anatomical linkages that serve to connect individual neural cells. This kind of structural characterization is clearly not irrelevant when it comes to understanding the functional dynamics of neural information processing—any form of structural organization (aside from a fully connected network) will serve to constrain the *types* of effective connectivity that the network can ultimately support.^10^ By itself, however, a connectomic description of the brain tells us very little about its information processing capacity. To see this, we need only consider the work that has been undertaken in respect of invertebrate nervous systems (Marder and Bucher 2007; Meyrand et al. 1994; Selverston 1995). Here, we see that the very same (structurally-static) neural circuit can be dynamically reconfigured or ‘rewired’ as a means of supporting multiple forms of effective connectivity. Again, just to be clear, it is not that the structural organization of the neural circuit is irrelevant: patterns of effective connectivity are always constrained by underlying structural specifications. However, what ultimately underlies the computationally-significant properties of a networked ensemble (e.g., the ability of a network to yield contextually-nuanced patterns of adaptive behavior) is the time-variant flow of information and influence within a multiplicity of effective circuits, each of which is dynamically assembled from the same statically-configured structural base.

How does all this relate to issues of collective cognition and mandevillian intelligence? To answer this question it helps to think about the way in which time-dependent changes in agent-level properties may work to dynamically alter the effective connectivity of a communication network in a manner that enhances the performance profile of the multi-agent ensemble. One such factor, for example, might be the level of trust that exists between individual agents. When agents have high trust in one another, we might expect them to exert greater levels of influence than in situations of low trust, and this is likely to mean that the density of information flow and influence is greater (or more rapid) in high trust situations. If true, this would hint at a potentially interesting hypothesis concerning the role of distrust in *enhancing* certain kinds of collective problem-solving performance (Smart et al. 2010). Because distrust may effectively retard the rate of information dissemination through a network, collective problem-solving performances may be enhanced in situations where agents initially distrust one another, or are at least somewhat circumspect about what others tell them. We can easily envisage a situation in which initially low levels of trust (at the outset of a collective problem-solving endeavor) are progressively supplanted by greater levels of trust as the agents learn to work together. Such a profile would clearly deliver, roughly, the same sort of pattern of information flow and influence as that seen in the dynamic network simulations reported by Smart et al. (2010).

Another factor of interest concerns the way in which a steadfast commitment to one’s own ideas, creations, methods, beliefs, etc., may be gradually eroded as the community begins to converge on what is (hopefully) the best solution. The basic idea is that it is relatively easy to persevere with your own (perhaps eccentric and irrational ideas) when there is a substantial degree of diversity in the larger population. As more and more individuals begin to converge on a single, preferred solution, however, it becomes progressively harder to resist the forces of social influence that are exerted by your network peers. The result, we may assume, is a time-dependent increase in the level of social influence that works to encourage convergence on a particular solution.^11^ Once again, the effective connectivity of the network that mediates this kind of collective response profile is not all that dissimilar from the sort of time-variant changes in network structure reported by Smart et al. (2010).

Interestingly, when it comes to issues of social influence, a number of authors have highlighted the role of an individual’s ‘irrational’ adherence to their own discoveries as a means of promoting diversity, encouraging exploration, and preventing premature convergence on sub-optimal solutions. Zollman (2010), for example, suggests that an individual’s tenacity in maintaining their grip on certain ideas and beliefs can help to produce transient forms of epistemic diversity that work to the overall good of a collective cognitive community. Such tenacity, Zollman (2010) suggests, could easily be seen as a form of intellectual dogmatism, which is typically denigrated as a cognitive vice (Baehr 2006; Battaly 2008). Similar views are expressed by Xu et al. (2016) following the results of multi-agent computer simulations in a search task similar to that studied by Lazer and Friedman (2007). Their results suggest that an irrational, dogmatic adherence to certain kinds of belief can function to enhance the collective search performance of an agent community. While, at the individual level, a resistance to social influence might appear highly irrational and epistemically pernicious, especially when seemingly better ideas are on offer, the fact that some individuals refuse to alter their own views appears (at least in these cases) to work to the overall good of the community.^12^ It is, in other words, at the *collective* level that we see the epistemic benefit of a seemingly sub-optimal *individual* cognitive strategy. In situations such as these, individual vices emerge as a potentially important part of the socio-cognitive machinery that makes certain kinds of collective intelligence materially possible.

What we end up with, therefore, is a claim about the functional significance of cognitively-relevant properties that are typically seen as sub-optimal, at least when it comes to some aspect of individual performance. In at least some cases, these properties may change over the course of problem-solving, yielding time-dependent changes in the effective connectivity of a communication network. And as a result of these changes, the collective cognitive dynamics of the larger systemic organization (e.g., the team, group or socio-technical system) may be influenced in such a way as to ensure enhanced levels of cognitive performance. This helps us understand the results of Zollman (2010) and Xu et al. (2016) with respect to the beneficial effects of individual cognitive vice (dogmatism and irrationality) on collective performance. The individual cognitive properties, in this case, are reshaping the effective structure of the communication network in a manner that befits the processing demands of a particular task. This kind of network-centric account is by no means the only way of understanding the mechanistic underpinnings of mandevillian intelligence; it is, however, an account that establishes an important link with work that seeks to explore the relationship between communication network structure and the performance profile of collective cognitive systems (Kearns 2012; Mason et al. 2008; Baronchelli et al. 2013).

## Collective creativity

For the most part, the computer simulation studies reviewed in previous sections relied on the use of agents that lacked any intrinsic cognitive processing capabilities. The agents in Lazer and Friedman’s (2007) study, for example, were little more than simplified processing units that could compare the value of currently adopted solutions with those of their network peers. These studies are useful in terms of their ability to highlight the ways in which psycho-cognitive and psycho-social processes could affect the performance profile of an agent community, but they are somewhat lacking when it comes to evaluating the notion of mandevillian intelligence. This is because the notion of mandevillian intelligence focuses on the relationship between cognitive properties at *both* the individual and the collective level. It is therefore important to consider studies that feature agents possessing some degree of cognitive sophistication, i.e., agents that have an ability to engage in cognitively-relevant information processing. By using agents with cognitive abilities (i.e., cognitive agents), we are in a much better position to systematically assess the interplay between forces and factors that operate at both the individual and collective levels.

One example of a study that uses computational agents with some degree of internal cognitive complexity is a study by Bhattacharyya and Ohlsson (2010). Bhattacharyya and Ohlsson (2010) were interested in the factors that influenced the creativity of social groups. In particular, they sought to explore the extent to which larger working memory capacities (defined as the number of information items that an agent could manipulate and transform at any given step of a simulation) could influence collective creativity. The task confronting the agents was one that involved the progressive transformation of a candidate solution, encoded as a symbol vector (e.g.,<0, 1, 9, 3, 8, 2, 7, 8, 4>), into a predefined target solution (e.g.,<0, 1, 2, 3, 5, 6, 7, 8, 9>).^13^ Each agent in the study was represented as a set of cognitive structures and processes. These included a long-term memory store (referred to as the “stock”), a capacity-constrained working memory store (referred to as the “active list”), a set of mechanisms for transferring information between long-term and working memory stores, and a set of operations for generating new candidate solutions (i.e., symbol vectors) from existing ones. Importantly, agents were only able to create new candidate solutions from the symbol vectors that were already contained in working memory. Thus agents with more limited working memory capacities (e.g., an active list constrained to hold five symbol vectors) would have a more restricted pool of candidate solutions to work with than agents endowed with larger working memory capacities (e.g., an active list constrained to hold fifteen symbol vectors). In addition, on each cycle of the simulation, agents were able to communicate information about a subset of the candidate solutions (again contained in working memory) to other agents in the simulation. This meant that agents could distribute the effort associated with the search for the target solution and benefit from the creative efforts of other agents. The candidate solutions selected for communication to other agents were determined by a noisy evaluation (or objective) function that indicated the relative distance of a specific solution candidate to the target solution. This information was also used by agents on each cycle of the simulation to select (1) the symbol vectors to be committed to long-term memory, (2) the symbol vectors to be retrieved from long-term memory, and (3) the symbol vectors to be modified on each cycle of the simulation. Importantly, only those symbol vectors that were contained in working memory could be manipulated by an agent. The working memory store of each agent was, in effect, the cognitive crucible from which new candidate solutions were forged.

Given that the agents in Bhattacharyya and Ohlsson’s (2010) study were only able to operate on the symbol vectors contained in working memory, we might expect groups of agents with higher working memory capacities to outperform groups of agents with lower capacities. Surprisingly, however, this is not what Bhattacharyya and Ohlsson observed. They discovered that when agents were situated in a communication network and were able to share the results of their individual creative endeavors with neighboring agents, an increase in working memory capacity did *not* improve the speed at which the community was able to discover the target solution. In fact, as working memory capacity increased from 10 to 20 vectors, Bhattacharyya and Ohlsson observed a slight reduction in the creative efficiency of the agent community (measured as the number of simulation cycles taken for at least one agent to discover the target solution). Limitations in the cognitive capacity of individual agents thus had the counterintuitive effect of improving collective cognitive outcomes.

In accounting for this pattern of results, Bhattacharyya and Ohlsson draw attention to the role of working memory constraints in focusing the attention of the agent community on a limited subset of high value candidate solutions. In essence, they suggest that by reducing the number of information items that individual agents are able to work with, the creative effort of the agent community ends up being restricted to those regions of the search space that contain the target solution:

A smaller active list [working memory] enables narrow focus on one target, while a larger active list tends to distribute the processing effort across a broader range of elements, some of which are not on the path to the target but nevertheless occupy memory space and processing cycles. (Bhattacharyya and Ohlsson 2010, p. 270)

Such interpretations are clearly compatible with the notion of mandevillian intelligence. When situated outside of a social context, we might be inclined to see the sort of capacity constraints described by Bhattacharyya and Ohlsson as reflecting something of a sub-optimal cognitive profile. Indeed, it might very well be the case that such constraints *undermine* the performance of individual agents when they engage in creative tasks independently of other agents. Higher working memory capacities might, for example, be a boon to creative efficiency when it comes to an examination of the performance of solitary individuals (e.g., a lone genius). Once situated within a social context, however, it appears that individual-level constraints in working memory might be deserving of a more positive interpretational gloss. Here, the capacity constraints seem to be shaping the creative efforts of the agent community in a manner that helps the community settle on the best solution in the most efficient manner. In particular, the results of Bhattacharyya and Ohlsson’s study seem to suggest that restrictions on individual working memory might contribute to a productive narrowing of communal attention, enabling an agent community to channel their collective creative resources into the exploration of those regions of the search space in which the globally-optimal solution is most likely to be found.

## Forgetting in a social context

The study by Bhattacharyya and Ohlsson (2010) serves as a useful example of a computer simulation experiment that features agents with a moderate degree of cognitive sophistication. A study that relies on the use of agents with even greater levels of cognitive sophistication and complexity is described by Reitter and Lebiere (2012). This study attempts to explore the relationship between individual-level mnemonic capabilities and collective performance on a spatial search task. One of the interesting features of Reitter and Lebiere’s study is the use of the ACT-R cognitive architecture (Anderson 2007; Anderson et al. 2004). In particular, the agents in Reitter and Lebiere’s study were implemented as separate ACT-R models, each of which attempted to emulate the performance of human subjects on a similar task. One of the advantages of using ACT-R in this kind of simulation context is that it provides access to a rich array of parameters that can be used to modify the cognitive performance profile of individual agent-level models. Using one such parameter—a parameter that affects the rate at which information in memory decays over time—Reitter and Lebiere were able to study the effect of individual mnemonic decay (i.e., forgetting) on aspects of collective performance. A particular focus of attention was the extent to which cognitively plausible rates of forgetting (i.e., the rates of forgetting that most resemble those of human subjects) could be of adaptive value in terms of influencing collective search behavior in a dynamically changing environment.

The specific task used by Reitter and Lebiere is called the Geo Game task. This is a spatial search task in which agents are presented with a map displaying cities connected by a road network. The goal of each agent is to travel between the cities and locate specific items, one at a time. When an agent finds a target item, they are assigned a specific number of points and are then given a new item to find. Importantly, whenever an agent reaches a city location they are shown a list containing *all* the items that are available at that particular location. This list might not contain the specific item that the agent wishes to locate; however, it might contain items that are the search targets of other agents. The community of agents can thus improve the efficiency of the collective search effort by sharing information with each other. As all the participants explore the spatial environment, they learn about the location of items, and by sharing this information, they help other agents find the items they need. Given that the transition from one city to the next incurs a time penalty, it benefits agents to request information from others and exploit any relevant knowledge that is communicated to them. For this reason, agents are able to request information from their immediate peers in a social network by posting simple messages, such as “need ladder.” Similarly, they are able to communicate information about the whereabouts of specific items by posting messages, such as “ladder in Moscow.” Agents are also able to re-transmit messages that they receive from other agents, thereby allowing messages to propagate throughout the entire agent community. The result is that agents are able to draw on the knowledge of other agents as a means of improving the efficiency of their own, individual search efforts.

The details of Reitter and Lebiere’s study are complex and a full description of their results is beyond the scope of the present paper. What is important for present purposes, however, is that by manipulating the rate at which information in an agent’s memory decayed over time, Reitter and Lebiere were able to examine the relationship between forgetfulness at the individual level and aspects of collective performance (e.g., the number of target items found by *all* the agents). Clearly, given that the aim of the Geo Game is to retrieve items from specific locations, an ability to recall the whereabouts of an item (based on previous messages) is a distinct advantage, and we might thus expect agents with low rates of mnemonic decay (low forgetfulness) to perform better than agents with higher rates of decay (high forgetfulness). Importantly, however, Reitter and Lebiere changed the location of items throughout the game. In particular, they recreated an item at a different location every time it was retrieved by an agent. Thus, if the target item was a ladder, then when the ladder was eventually found by an agent in Moscow, it would be recreated in a different city—Berlin, for example. This manipulation thus served to implement a form of dynamic ground truth for the Geo Game: existing facts were continuously invalidated as the game progressed, and agents were thus required to continually update their knowledge about the location of relevant items. In this situation, the ability to recall all previously encountered facts starts to look unnecessary and perhaps a little counterproductive. In particular, many of the facts that are memorized by agents will fail to predict the location of target items as the time between encoding and recall increases. There is thus a risk that agents may recall outdated information that then leads their individual search efforts astray. In such situations, we might be inclined to say that the decay of information in memory is an adaptive response to the dynamic and turbulent nature of the problem-solving environment. In particular, by forgetting previously encountered information, agents are able to rid themselves of information that is of little predictive relevance within a dynamically changing search environment. This ability to forget outdated information is of adaptive value precisely because it enables an agent to link response output to those mnemonic representations that are the most recent (and thus the most reliable) sources of task-relevant information.

Such intuitions are supported by the results of Reitter and Lebiere’s study. They found that search performance tended to peak at intermediate levels of mnemonic decay. In addition, they discovered that agent communication benefited performance, but only when the rate of mnemonic decay was set at an intermediate level. For lower and higher rates of decay, the benefits of socially-mediated informational exchanges appeared to be reduced.^14^ Interestingly, the decay rates that yielded the greatest levels of collective success were those that are typically used to model human memory (see Anderson 2007, p. 110). This raises the intriguing possibility of what Reitter and Lebiere refer to as an “evolutionary co-optimization between the human cognitive system and an external social structure” (p. 1). The idea, in essence, is that the dynamics of human forgetting might be adaptively tuned to reflect the structure of the human social environment. As a result of this adaptive tuning, natural limitations in human memory could be seen as playing a productive role in yielding *collective* forms of cognitive success.

Reitter and Lebiere’s study is a compelling demonstration of the use of a popular cognitive architecture (i.e., ACT-R) to investigate the relationship between individual-level cognitive properties and the dynamics of collective performance. It is, moreover, a study that yields a set of empirical results that provide a degree of evidential support for the notion of mandevillian intelligence. As with the other studies reviewed in the present paper, Reitter and Lebiere’s study helps us see *how* an individual-level cognitive shortcoming (i.e., forgetting) could be of adaptive value in terms of supporting collective search efforts.^15^

## Confirmation bias and the cognitive division of labor

Aside from work into cognitive constraints and limitations, the notion of mandevillian intelligence also finds theoretical and empirical support from research into cognitive biases. Consider, for example, one of the most well-known forms of cognitive bias: confirmation bias (Nickerson 1998). Confirmation bias is generally regarded as the tendency for individuals to seek confirmatory evidence for existing beliefs and hypotheses, coupled with the failure to consider competing or contradictory evidence. This tendency has long been known to be a feature of our human cognitive character,^16^ and it is almost universally denigrated on account of its potential threat to our truth-seeking efforts. In particular, confirmation bias seems to represent a departure from the traditional image of the rational cognizer who engages in the objective evaluation of available evidence. Based on this interpretation, it is easy to see how confirmation bias might be seen as inimical to cognitive performance. It is for this reason, no doubt, that the minimization or elimination of confirmation bias has been the focus of so much attention by the human factors community (e.g., Convertino et al. 2008).

A more positive view of confirmation bias is provided by Mercier and Sperber (2011) as part of their attempt to explore the (biological) function of human reasoning. Mercier and Sperber challenge the conventional idea that the function of human reasoning is to provide a means by which we humans are able to discover and track the truth. Instead, Mercier and Sperber suggest that human reasoning is concerned with the production and evaluation of arguments that are used in the context of persuasive communications. The result is what Mercier and Sperber refer to as an Argumentative Theory of Reasoning (ATR)—a theory that emphasizes the role of reasoning as a form of “argumentative device.”

One of the advantages of the ATR, Mercier and Sperber claim, is that it helps us gain a better understanding of confirmation bias. If the purpose of human reasoning is to devise arguments that are intended to persuade, then it becomes much clearer why we humans might be inclined to seek out information that is consistent with and supportive of our preferred views and prevailing opinions. In contrast to the conventional view of confirmation bias as a form of reasoning gone wrong, the ATR suggests that confirmation bias is a perfectly natural feature of human reasoning. Confirmation bias, Mercier and Sperber suggest, emerges as “a consequence of the function of reasoning and hence [it is] a feature of reasoning when used for the production of arguments” (Mercier and Sperber 2011, p. 63).

This view of confirmation bias as a natural feature of human reasoning then leads Mercier and Sperber to make a rather radical claim. They propose that confirmation bias can actually enhance the efficiency of collective (group-level) problem-solving efforts:

When a group has to solve a problem, it is much more efficient if each individual looks mostly for arguments supporting a given solution. They can then present these arguments to the group, to be tested by the other members...This joint dialogic approach is much more efficient than one where each individual on his or her own has to examine all possible solutions carefully. The advantages of the confirmation bias are even more obvious given that each participant in a discussion is often in a better position to look for arguments in favor of his or her favored solution... (p. 65)

This quotation hints at a view of confirmation bias that is in perfect accord with the notion of mandevillian intelligence. In particular, when we look at confirmation bias through the lens of mandevillian intelligence, we can begin to see how confirmation bias might work to the overall good of a community of truth-seeking agents. In order to help us see this, imagine that we have a group of individuals who are trying to navigate some complex problem domain. Providing that we have a sufficient degree of cognitive diversity (represented as differences in beliefs, opinions, theories, approaches, or whatever) at the outset of the problem-solving endeavor, then we can assume (in view of the ATR) that each agent will attempt to marshal support for their own argumentative position. Given that cognitive assets (as well as other resources, such as time) are invariably limited, it helps if each individual agent engages in a thorough exploration of a specific region of the problem space rather than have *all* agents attempt to engage in a systematic exploration of the entire space. Not only is this latter strategy likely to be infeasible, it is also likely to be ineffective: each agent, we may assume, will only be able to process the totality of information at a rather superficial level. In contrast, when each agent is attempting to bolster support for their own argument they will be encouraged to restrict their attention to a much more limited set of information (specifically, that information that supports their own views and opinions) and process this information to a much greater depth than would otherwise be the case—the agents are, after all (according to the ATR), attempting to construct a persuasive and convincing argument. By drawing on the notion of confirmation bias, we can plausibly infer that each agent will be inclined to actively discount, ignore or fail to search for information that conflicts with their initial view. This will obviously lead them to have a very selective view of the problem space. And, in many cases (e.g., where there is a single correct answer to a problem), we can assume that the vast majority of agents will be pursuing lines of enquiry that are ultimately unproductive. When seen from a normative epistemological perspective, the behavior of the individual agents in this situation looks far from optimal, and this is particularly so when we look at the situation from the perspective of individualistic epistemology. In this case, we might be inclined to see the individual agents as epistemically defective relative to some normative standard of epistemic virtue. Things, however, look rather different when we approach matters from the perspective of mandevillian intelligence. In this case, the epistemic shortcomings of the individual play a positive role in helping the community to organize its collective efforts in an epistemically propitious manner. It is, in fact, the cognitive vices of the individual agent (cashed out in terms of the agent’s susceptibility to confirmation bias) that serves as the basis for the expression of virtuous characteristics that then get ascribed to the larger socio-epistemic organization. If each agent was, in fact, to behave in a perfectly rational, objective and impartial manner, then it seems far from clear that the collective, as a whole, would be able to make sufficient progress on the matter at hand. We might, for example, expect each and every agent to fail to find the truth on account on their superficial coverage of the relevant information space, perhaps because each agent attempts to rigorously evaluate all available information. In this situation, it seems much better to organize the community so that individual agents will focus on distinct and somewhat limited parts of the problem space, marshal arguments in favor of their particular position, and then attempt to recruit as many followers as possible. It is at this point, as Mercier and Sperber (2011) are keen to stress—the point at which we attempt to convince others of our own positions—that we approach something resembling the classic conception of reason and rationality: during the process of group discussion, ideas are subjected to critical evaluation, flaws are identified, and the truth, hopefully, is at last revealed.

When it comes to the realm of rational thought, therefore, it appears that we ought to locate our epistemically-optimal (i.e., truth-conducive) ratiocinative capabilities at the collective (as opposed to the individual) level. It is, moreover, unclear that the epistemic interests of the many are always best served by the elimination of (individual forms of) confirmation bias. In our attempts to make each individual the perfect cognizer, we may inadvertently undermine our collective ability to probe a space of epistemic possibilities and converge on those places where the truth is most likely to be found.

## Implications of mandevillian intelligence

We have now explored a number of lines of empirical research that provide support for the notion of mandevillian intelligence, i.e., the claim that individual cognitive vices can, on occasion, work to the collective cognitive and epistemic good of an agent community. But what, if any, bearing does this have on existing debates regarding collective cognition and collective intelligence? How, in particular, does the notion of mandevillian intelligence affect our understanding of extant socio-cognitive ensembles? And does the notion of mandevillian intelligence provide any insight into how we might enhance collective cognitive capabilities via the development of collaborative technologies? What then is the practical value of the concept of mandevillian intelligence in philosophical, scientific and engineering terms? In this section, I attempt to outline a number of initial responses to these questions.

### Augmented cognition?

When it comes to issues of cognitive augmentation, one thing seems relatively clear: inasmuch as we accept the notion of mandevillian intelligence, we will need to pay much closer attention to the relationship between properties at the individual and collective level. In the absence of the concept of mandevillian intelligence, we might be misled into thinking that the route to collective intelligence always has its origins in the enhancement of individual cognitive functioning. The notion of mandevillian intelligence, however, helps to promote a degree of caution here. In particular, it opens up the possibility that by enhancing the cognitive profile of the individual agent, we may inadvertently undermine the performance profile of the larger collective organization (e.g., socio-technical system) in which that individual is situated. There is, therefore, something of a potential tension when it comes to the enhancement of individual and collective cognition. In particular, we should not assume that an intervention that improves the cognitive functioning of the individual agent will *always* lead to beneficial effects at the collective level. There may, indeed, be cases where the attempt to enhance individual cognition undermines, or at least destabilizes, collective performance.

These considerations are relevant to contemporary debates regarding human cognitive augmentation and enhancement. Although considerable interest has been expressed in the use of (e.g.) technological and pharmacological interventions to improve human cognitive performance (e.g., Zonneveld et al. 2008; Bostrom and Sandberg 2009), the notion of mandevillian intelligence forces us to question whether these effects obtain at both the individual and the collective levels. In fact, with the notion of mandevillian intelligence to hand, it now seems possible that individual forms of cognitive enhancement may, on occasion, come at the expense of collective forms of cognitive diminishment. The concept of mandevillian intelligence thus provides the basis for future research that seeks to explore the nature of the trade-offs between individual and collective forms of cognitive augmentation, as well as the kinds of conditions under which these trade-offs occur.

### Virtuous engineering?

Another issue that the notion of mandevillian intelligence helps to bring into sharper focus is the idea that cognitive limitations and biases at the individual level can be deliberately exploited, or even amplified, as a means of improving collective performance. This is clearly an issue that is likely to raise a host of ethical concerns, and not everyone, I suspect, will be inclined to embrace it. Nevertheless, the idea of using technologies to allow or promote seemingly sub-optimal forms of individual behavior is not new. March (2006), for example, argues that the use of technologies to encourage irrational behavior can sometimes be of benefit to an organization. He suggests that “a technology of rationality has to be balanced by other technologies that free action from the constraints of conventional knowledge and procedures and introduce elements of foolishness into action” (March 2006, p. 203).

In order to help us get better grip on what is being proposed here, consider the way in which mandevillian intelligence forces us to question our intuitions as to the functional role of individual cognitive biases in collective performances. In the case of confirmation bias, for example, we noted that (individual) confirmation bias is not something that is to be unconditionally reviled; in certain situations, confirmation bias may play a positive role in enabling a community of agents to achieve sufficient coverage of a space of possible solutions, perhaps by preserving and reinforcing diverse lines of enquiry. The effect of all this is to challenge our sense as to what a technology for collective intelligence ought to do. Typically, we tend to think that our technology design efforts should be guided by the goal of removing or minimizing individual forms of cognitive bias. But once we acknowledge the basic possibility of mandevillian intelligence, we are in a position to contemplate an alternative approach to technology design. In this case, the aim becomes not so much the mitigation of individual forms of cognitive vice, as the exploitation (or exacerbation) of such vices for the purposes of enhancing collective forms of intelligence.

In support of this rather counter-intuitive (and undoubtedly contentious) claim, notice that one of the enduring findings to come out of group performance research is the idea that cognitive diversity is, in general, a boon when it comes to collective forms of cognitive success (Schulz-Hardt et al. 2000; Convertino et al. 2008; Hong and Page 2004). In their attempt to evaluate a collective sensemaking technology, for example, Convertino et al. (2008) began by exposing group members to evidence favoring different hypotheses. The purpose of this manipulation was to artificially instill a degree of cognitive diversity between the individual problem-solving agents. This manipulation, as it turned out, was useful in enabling the groups to settle on the correct answer to the sensemaking problem. But why, we might ask, should this sort of manipulation (selective exposure to particular bodies of information) not be a feature of technologies that are used beyond the laboratory environment. In other words, why not enable sensemaking technologies to deliberately create the kinds of conditions that enable the technology to be used to its greatest effect? At the outset of a sensemaking task, a sensemaking technology could thus engage in the active manipulation of information flow patterns in ways that seek to establish the (initial) cognitive diversity that is relevant to subsequent forms of collective cognitive success. One way this could be achieved is by limiting the access that individuals have to particular bodies of information, as well as by carefully monitoring (and actively managing) the flow of information between individual agents. As a result of this deliberate attempt to establish cognitive diversity, we could easily imagine our apparent tendency to filter information on the basis of our initial convictions (i.e., confirmation bias^17^) being pressed into useful productive service, encouraging agents to embark on the independent exploration of distinct trajectories through a space of interpretational possibilities.

What all this amounts to, I suggest, is an approach to technology development that factors in the potential contribution of individual cognitive biases and processing limitations as a means of enhancing collective cognitive performance. The general idea is that the cognitive vices of the individual are an exploitable resource that, when situated in the right kind of socio-technical context, can work to the overall cognitive good of a collective, multi-agent ensemble. The properties of the individual are, if you like, part of a suite of mechanisms that technology developers can rely on as part of their attempt to design and develop socio-cognitive or socio-computational systems.

### Vicious technology?

Finally, notice how the notion of mandevillian intelligence influences the nature of debates concerning the cognitive and epistemic impacts of particular technologies. As an example, consider one of the major areas of epistemological enquiry into the World Wide Web: the use of Internet search engines, such as Google Search (Miller and Record 2013; Simpson 2012). A particular focus of attention in this debate concerns the impact of personalized search mechanisms on the epistemic credentials of the human individual. Epistemologists are largely in agreement regarding the negative effects of such mechanisms. Simpson (2012), for example, argues that personalized search accentuates the problem of confirmation bias and undermines users’ access to objective information. Similar views are espoused by Miller and Record (2013) who claim that the justificatory status of an agent’s beliefs are undermined as a result of exposure to personalized search results. Personalized search, they suggest, presents individuals with information that is often biased and incomplete, and there is no reason, they claim, to think that the operation of personalized search algorithms establishes any sort of productive alignment with epistemic desiderata such as reliability, objectivity, scope and truth.

The arguments of Miller, Record and Simpson appear, on the surface at least, to be perfectly plausible. They thus invite speculation as to how we should seek to minimize the negative epistemic effects of personalized search. Among these recommendations we encounter a proposal for governments to legally oblige search providers to behave in an epistemically optimal (or at least benign) manner (Simpson 2012). The primary concern, here, is that personalized search is a threat to the objectivity of epistemic agents, and given that objectivity is seen as publicly desirable—a public good—the government regulation of search engine providers seems to be an appropriate and prudent course of action.

Should we, however, be so quick to accept the claims of Miller, Record and Simpson regarding the epistemic sequelae of personalized search engines? The problem is that once we acknowledge the existence of mandevillian intelligence, it looks as though something may have been overlooked in the epistemological debate. Given the claim that public (collective) virtue can sometimes derive from private (individual) vice, we might wonder whether the introduction or accentuation of a private vice should always be condemned in the absence of some broader understanding of its effects at the social or collective level. The problem is that even if we accept the claim that personalized search is having a negative effect on our individual epistemic profiles, it is far from clear that it is *also* having a negative effect on our collective epistemic capabilities. In fact, in view of the earlier discussion about the role of confirmation bias in supporting the cognitive division of labor (see Sect. 7), there seems to be at least a prima facie case for thinking that it is in fact a *virtue* of personalized search technologies that they manage to influence and shape a user’s search efforts in the sort of way that Miller, Record, Simpson and others^18^ find so disagreeable.

One way to understand the potentially positive effects of personalized search is in terms of the technological accentuation of confirmation bias and selective information exposure processes. Such processes, we have seen, play a potentially useful role when it comes to the effective exploration of a complex space of ideas, discoveries, and interpretational possibilities. In the case of the ATR, for example, Mercier and Sperber (2011) discuss the role of confirmation bias in yielding a particular form of collective success based around individual search and communal evaluation. It is important to note, however, that the potentially positive role of personalized search should *not* be interpreted solely in terms of the ATR. This is because the kinds of discursive interaction and argumentative exchange alluded to by Mercier and Sperber (2011) are unlikely to apply in the specific case of the Internet and World Wide Web—the place where many forms of personalized search are likely to be encountered. Not only is communication between cognitively distinct sub-groups likely to be weak or non-existent in an online context, but the number of people involved (perhaps thousands) is unlikely to be conducive to conventional forms of collective debate and communal evaluation—at least of the sort envisaged by Mercier and Sperber. This does not mean, however, that the (collective) benefits of personalized search are always out of reach in an online context; it simply means that we need to envisage alternative ways of integrating and evaluating the results of individual search efforts. There are, in fact, a number of possibilities here. One is that an external agency could exploit the independent cognitive efforts of distinct communities for their own purposes. An intelligence agency, for example, might opt to monitor the activity of multiple groups within distinct social media systems as a means of supporting their own sensemaking efforts (see Nhan et al. 2017). The operation of personalized search (and the presence of echo chambers), in this situation, could help to maintain cognitive diversity between distinct sub-groups of ‘citizen sensemakers’, each of which works on a particular aspect or dimension of a larger sensemaking problem.^19^

A consideration of mandevillian intelligence thus presents us with an expanded view of the epistemic (and cognitive) impacts of a particular technology. As should by now be clear, the veritistic value of a technology may vary depending on whether we choose to focus our attention at the individual or collective level. While a technology may work to undermine the epistemic profile of an individual, this does not mean that the same technology will also undermine the epistemic capabilities of a collection of such individuals. In fact, it may very well be the case that the technology-mediated accentuation of an individual shortcoming (e.g., the aforementioned accentuation of confirmation bias) will play a positive functional role in ensuring optimal performance at the collective level. As a result, even if we choose to accept the claims of Miller, Record and Simpson, it is difficult to determine whether the kind of remedial strategies (e.g., government intervention) they propose are warranted, justified, or even desirable. It is quite possible that by attempting to attenuate the negative epistemic impacts of personalized search at the individual level we will inadvertently jeopardize the opportunities for epistemic progress at the collective level. In this regard, it is interesting to note that in emphasizing the impact of personalized search on the public good, Simpson (2012) makes the implicit assumption that public goods derive from individual objectivity, or that objectivity is, in itself, a public good. The notion of mandevillian intelligence provides us with a new perspective on this debate. In particular, it enables us (and perhaps obliges us) to consider the extent to which the public good (e.g., the ability of a community to fathom the truth) is not only served by but also perhaps dependent upon the presence of individual vice. Far from harming the public good, personalized search may be just what is required in order for social groups (and society at large) to reap the epistemic benefits of the online, digital world.

## Conclusion

Mandevillian intelligence is the idea that individual-level cognitive shortcomings, biases, limitations and constraints can, on occasion, give rise to collective-level benefits. The present paper has sought to review a number of strands of evidence that provide support for this idea. We have thus examined the results of studies that indicate a potentially positive role for factors like dogmatism (Xu et al. 2016; Zollman 2010), confirmation bias (Mercier and Sperber 2011) and mnemonic constraints (Bhattacharyya and Ohlsson 2010; Reitter and Lebiere 2012) in supporting the performance of teams, groups and communities.

There is, of course, much more philosophical and scientific work to be done in respect of the notion of mandevillian intelligence. Of particular importance, is the need to investigate the explanatory and predictive value of the concept with respect to *human* collectives. It will thus be important, in future work, to examine the conditions under which individual forms of cognitive vice yield positive effects on collective processing in human teams, groups and communities. Such work presents us with a host of ethical and methodological issues, since it is not always possible (or desirable) to manipulate individual cognitive functioning in the sort of way required to experimentally evaluate claims of mandevillian intelligence (e.g., deliberately reducing an individual’s attentional or mnemonic capabilities). One possibility is exemplified by Hourihan and Benjamin (2010): exploit the naturally-occurring inter-individual variability that exists with respect to some cognitive parameter of interest. Another approach, perhaps, is to rely on the use of manipulations, such as dual-task procedures (e.g., Olive 2004), that temporarily interfere with some aspect of task-relevant performance.

As with any concept, the notion of mandevillian intelligence earns its ontological keep to the extent that it provides us with a better explanatory and predictive grasp on some target set of phenomena. In the case of mandevillian intelligence, this is obviously something that should be judged in the light of future empirical work. A concept is also valuable to the extent that it affords a novel perspective on the world—one that highlights features that would otherwise be invisible, and one that encourages us to ask questions that we would otherwise fail to ask. In this respect, we have seen that mandevillian intelligence can help to inform debates regarding the cognitive and epistemic significance of existing technologies. The general lesson, here, is that the cognitive and epistemic value of a specific technological intervention (e.g., personalized search) may vary according to whether our evaluative gaze is oriented towards the individual or collective level.

The concept of mandevillian intelligence also helps to inform debates concerning the attempt to enhance collective cognition. From the perspective of mandevillian intelligence we can begin to think about the trade-offs in individual and collective performance that might occur as the result of some form of technological, pharmacological, or organizational intervention. We can also begin to wonder whether, in the context of our technology design efforts, an individual cognitive shortcoming should always be viewed as problem to be remedied, as opposed to an opportunity to be exploited. The thing to remember, here, is not that these questions will always pave the way to improvements in collective performance—in many cases, we may very well discover that individual cognitive vices do not, in fact, deliver an improvement in collective cognizing. The point is that without some form of conceptual underpinning, these sorts of questions might never be asked or taken seriously. What the notion of mandevillian intelligence gives us, I suggest, is an argumentative foundation for new ideas that enable us to challenge received wisdom and orthodox practice. Some of these ideas may be contentious; others may very well prove invalid or unworkable in practice. This does not, however, undermine the value of the concept of mandevillian intelligence in terms of its ability to stimulate scientific and philosophical debate, question long-held assumptions, open up new avenues for research and (above all) provide us with a new way to think about how the properties of the individual mind may shape the course of our collective cognitive endeavors.

## Footnotes

1 The term “collective cognition” is used here as an umbrella term for forms of cognition that involve multiple (autonomous) agents. Examples include group cognition (Theiner 2014), team cognition (Cooke et al. 2007), distributed cognition (Hutchins 1995), swarm cognition (Trianni et al. 2011) and, of course, collective cognition (Couzin 2009).

2 Some examples of negative character traits (dubbed intellectual vices) include dogmatism, closed-mindedness and gullibility. These contrast with a collection of positive character traits, such as conscientiousness, curiosity, objectivity and impartiality, which are said to qualify as intellectual virtues (see Battaly 2008).

3 Consider, for example, the case where a law enforcement agency wishes to subvert the decision-making capabilities of a terrorist group or criminal fraternity.

4 An alternative, although not altogether unrelated, view of cognitive vice and virtue derives from virtue epistemology (Greco and Turri 2012). In this case, the notions of vice and virtue are defined relative to the reliability of a cognitive property, where reliability is understood in terms of the generation of true beliefs. From a virtue epistemological perspective, therefore, all cognitive biases count as vices because they undermine the chances that an individual will believe the truth. A virtue, in contrast, is any cognitive property (e.g., a cognitive ability) that leads to a preponderance of true beliefs.

5 The nature of the objective function typically varies based on the goals of a particular experiment. In many studies, for example, agents do not see the actual value of the objective function; instead, they are provided with noisy feedback. In other studies, the objective function changes across time, yielding time-variant deformations in the topological structure of the solution landscape. Such deformations occur as the result of exploration efforts or as the result of dynamic changes in the search environment. Objective functions may also vary between agents, with some subset of agents trying to solve separate, but nevertheless interdependent, parts of a larger, more complex problem.

6 Exploration involves the attempt to expand what is currently known, while exploitation concerns the processes associated with the refinement and implementation of what is currently known. March (2006) suggests that “Exploitation involves the application of established competence to problems” (p. 205), while “Exploratory activities are sources of novel, unconventional, improbable, wild ideas and actions” (p. 205).

7 Note that the role played by these cognitive properties could be fulfilled by any number of functionally-equivalent interventions. We could, for example, imagine so-called “social network incentives” (see Pentland 2014) being used to shape the actual flow of information during the course of problem solving. We can also begin to see how unwanted forms of cognitive convergence could be prevented (or at least postponed) by access policies, security constraints, limited information exchange and interoperability capabilities, copyright legislation, and so on. The point to bear in mind, here, is that the functional role played by a particular kind of socio-technical or organizational intervention could also, at least in principle, be fulfilled by some aspect of agent-level cognitive functioning (e.g., a form of biased processing, a cognitive constraint, or a seemingly undesirable feature of an agent’s cognitive character).

8
Lazer and Friedman (2007) evaluated the impact of copying errors in their computer simulation studies. They report that, in the long-run, systems with higher error rates in the copying process outperformed those in which copying errors were minimized. The explanation for these results is the same as that proposed for the effect of network structure on performance, namely, that higher rates of error alter the balance between exploration and exploitation, increasing the amount of exploration but reducing the rate at which superior solutions spread.

9 Interestingly, Couzin (2009) suggests that we can see these pheromonally-mediated behaviors as part of the physical realization base for cognitive processes that then get ascribed to the larger collective organization. He thus suggests that “The separation of timescales afforded by employing chemicals with different volatilities enables a form of ‘working memory’ (encoded using slowly decaying chemicals, which if not reinforced, is eventually lost) and also a ‘selective attention’ (through the deployment of short-lived, but behaviorally dominant chemicals) in which relevant events, such as exploitable resources, become the focus of foraging activity” (p. 41). Inasmuch as we want to see chemical trails as a form of external memory, then it is possible to regard the dissipative properties of pheromonal signals as a form of mnemonic decay (or forgetting). Consistent with the overall tenor of the present paper, this feature (i.e., mnemonic decay) plays an important (and perhaps indispensable) role in the adaptive coordination of collective behavior. Absent the natural tendency of chemical signals to decay over time and the ability of ants to make collectively-optimal decisions might be severely compromised. In particular, it might be extremely difficult for ant colonies to allocate food foraging assets (i.e., individual ants) in ways that are optimally aligned with the temporal availability and relative profitability of different food sources.

10 A fully connected network can obviously provide the structural base for any form of effective connectivity. It can thus support the emergence of *effective* network structures that correspond to small-world, scale-free, or random networks.

11 Such processes are thought to be a common feature of collective decision-making in a number of animal species (Couzin 2009). A key function of such forms of “graded recruitment,” Couzin (2009) suggests, is that they enable “a relatively slow, but more accurate, phase of decision-making to begin with and, later, a fast committed phase to decrease the overall time taken to make the decision” (p. 41).

12 It is important to note that dogmatism need not benefit performance simply because the dogmatist has (perhaps accidentally) hit on the right solution. Rather, what is important is that adherence to a particular set of ideas works to maintain a degree of cognitive diversity in the population as a whole.

13 According to Bhattacharyya and Ohlsson (2010), these symbol vectors correspond to the mental representations and/or physical artefacts that are generated by a creative process. Symbol vectors, they suggest, should be seen to stand for “ideas, concepts, data, questions, theories, propositions, schemas, beliefs, blue prints, tactics, plans, goals subgoals and so on” (p. 266).

14 These results were obtained following the removal of statistical outliers from the original dataset. This meant that 17% of the high performing agents were excluded from the analysis. When these high performing agents were included in the analysis, the number of retrieved items was greatest in conditions with the lowest decay value (i.e., the least forgetful agents). It thus appears that one effect of minimizing forgetfulness is to introduce higher levels of variability in performance, with a minority of agents enjoying superior levels of search success relative to their peers. The reason for this elevated variability is unclear at the present time.

15 In particular, the notion of mandevillian intelligence helps to highlight the role of cognitive vices as part of the physical machinery (or mechanistic substrate) that realizes (successful) episodes of collective cognizing.

16 Francis Bacon (2000), for example, noted that “Once a man’s understanding has settled on something (either because it is an accepted belief or because it pleases him), it draws everything else also to support and agree with it. And if it encounters a larger number of more powerful countervailing examples, it either fails to notice them, or disregards them, or makes fine distinctions to dismiss and reject them, and all this with much dangerous prejudice, to preserve the authority of its first conceptions” (p. 43).

17 The productive use of anchoring is another possibility here. Anchoring is a cognitive bias that emerges as the result of a tendency to over-commit to explanations or hypotheses based on the initial exposure to particular subsets of information (see Tversky and Kahneman 1974).

18 Consider, for example, recent debates relating to so-called “echo chambers” and “filter bubbles” (Pariser 2011).

19 Another possibility is that the nature of the task is such that a correct solution is easily recognized as soon it is discovered. In this case, personalized search algorithms may help to preserve diversity, but once a discovery is made, everyone is happy to accept that the correct solution has been discovered without the need for further discussion.
